# Linked-read sequencing for detecting short tandem repeat expansions

**DOI:** 10.1038/s41598-022-13024-4

**Published:** 2022-06-07

**Authors:** Readman Chiu, Indhu-Shree Rajan-Babu, Inanc Birol, Jan M. Friedman

**Affiliations:** 1 Canada’s Michael Smith Genome Sciences Centre, BC Cancer, Vancouver, BC V5Z 4S6 Canada; 2 Department of Medical Genetics, University of British Columbia, Vancouver, BC V5Z 4H4 Canada; 3 Department of Medical and Molecular Genetics, King’s College London, Strand, London, WC2R 2LS UK; 4 BC Children’s Hospital Research Institute, Vancouver, BC V5Z 4H4 Canada

**Keywords:** Computational biology and bioinformatics, Genome informatics

## Abstract

Detection of short tandem repeat (STR) expansions with standard short-read sequencing is challenging due to the difficulty in mapping multicopy repeat sequences. In this study, we explored how the long-range sequence information of barcode linked-read sequencing (BLRS) can be leveraged to improve repeat-read detection. We also devised a novel algorithm using BLRS barcodes for distance estimation and evaluated its application for STR genotyping. Both approaches were designed for genotyping large expansions (> 1 kb) that cannot be sized accurately by existing methods. Using simulated and experimental data of genomes with STR expansions from multiple BLRS platforms, we validated the utility of barcode and phasing information in attaining better STR genotypes compared to standard short-read sequencing. Although the coverage bias of extremely GC-rich STRs is an important limitation of BLRS, BLRS is an effective strategy for genotyping many other STR loci.

## Introduction

Barcode linked-read sequencing (BLRS) technologies^1–3^ combine the high per-base accuracy of short-read sequencing (SRS) with long-range sequence information^4^. BLRS has significantly advanced our ability to map complex genomic regions that are inaccessible to standard SRS, perform de novo diploid genome assemblies, and detect complex structural rearrangements^4,5^. BLRS enables haplotype reconstruction and accurate variant phasing^1,6^, which are crucial to identifying putative disease-causing biallelic mutations. BLRS also has been successfully applied to diagnose patients with suspected genetic diseases and failed diagnosis using exome/genome SRS^4^.

The human genome harbors over a million short tandem repeats (STRs)^7^. STR expansions are responsible for at least 50 known genetic disorders, and others probably still remain to be discovered^8^. Available computational methods^9–14^ and pipelines^15^ for STR genotyping in SRS data perform reasonably well in detecting pathogenic repeat expansions (REs) of some disease genes. Detection of in-repeat reads (IRRs)—reads composed entirely of repeat motif sequences—signal a potential RE event, and their abundance correlates with expansion size. However, accurate alignment of IRRs in SRS is often difficult because these reads may either not map to the reference genome or map ambiguously to multiple STR loci with the same repeat motif. As a result, existing short-read STR genotypers may report false-positive as well as false-negative calls for STRs that are larger than sequencing fragment length^15^.

We hypothesized that the molecular barcodes in BLRS can be utilized to retrieve IRRs more robustly and improve repeat length estimation and detection of expanded STRs. Furthermore, using barcodes could help assign IRRs to the correct haplotype and enable reliable allele segregation and genotyping of pathogenic biallelic STR expansions. To leverage this information, we devised a novel BLRS STR genotyping algorithm that uses the theoretical relationship between barcode sharing across genomic intervals and interval sizes.

We analysed data from three different BLRS methods—10 × Genomics Chromium, MGI stLFR, and Universal Sequencing Technology TELL-Seq. The BLRS datasets we analysed include (1) simulated 10 × and stLFR whole-genome sequencing (WGS) data containing a heterozygous 4000 ATTCT RE in the *ATXN10* gene; (2) publicly-available NA12878 Genome in a Bottle (GIAB) data from 10x, stLFR, and TELL-Seq BLRS platforms with a 1.1 kilobase (kb) expansion (relative to the reference) of a CCAT-repeat in chromosome 20; and (3) four 10 × WGS datasets with *FXN* GAA RE and four with *FMR1* CGG RE (see supplementary information). We benchmarked our approaches against ExpansionHunter (EH)^10,14^, the most widely-used short-read STR analysis tool, on BLRS and standard Illumina sequencing data (see supplementary information). To our knowledge, our study is the first to assess the utility of BLRS in detecting and genotyping STR expansions.

## Results and discussion

EH has not been evaluated on BLRS before. We ran EH (v2.5.5) on the BLRS datasets with or without off-target sites (OTS)—STR loci in the reference genome where IRRs containing the same repeat motif may have been mismapped (see supplementary information). Although helpful in detecting and accurately genotyping some large REs in SRS data^15^, using OTS may result in overestimation of repeat lengths and generate ambiguous genotype calls when an individual has more than one “expanded” STR with a shared repeat motif (data not shown).

**Figure 1:**
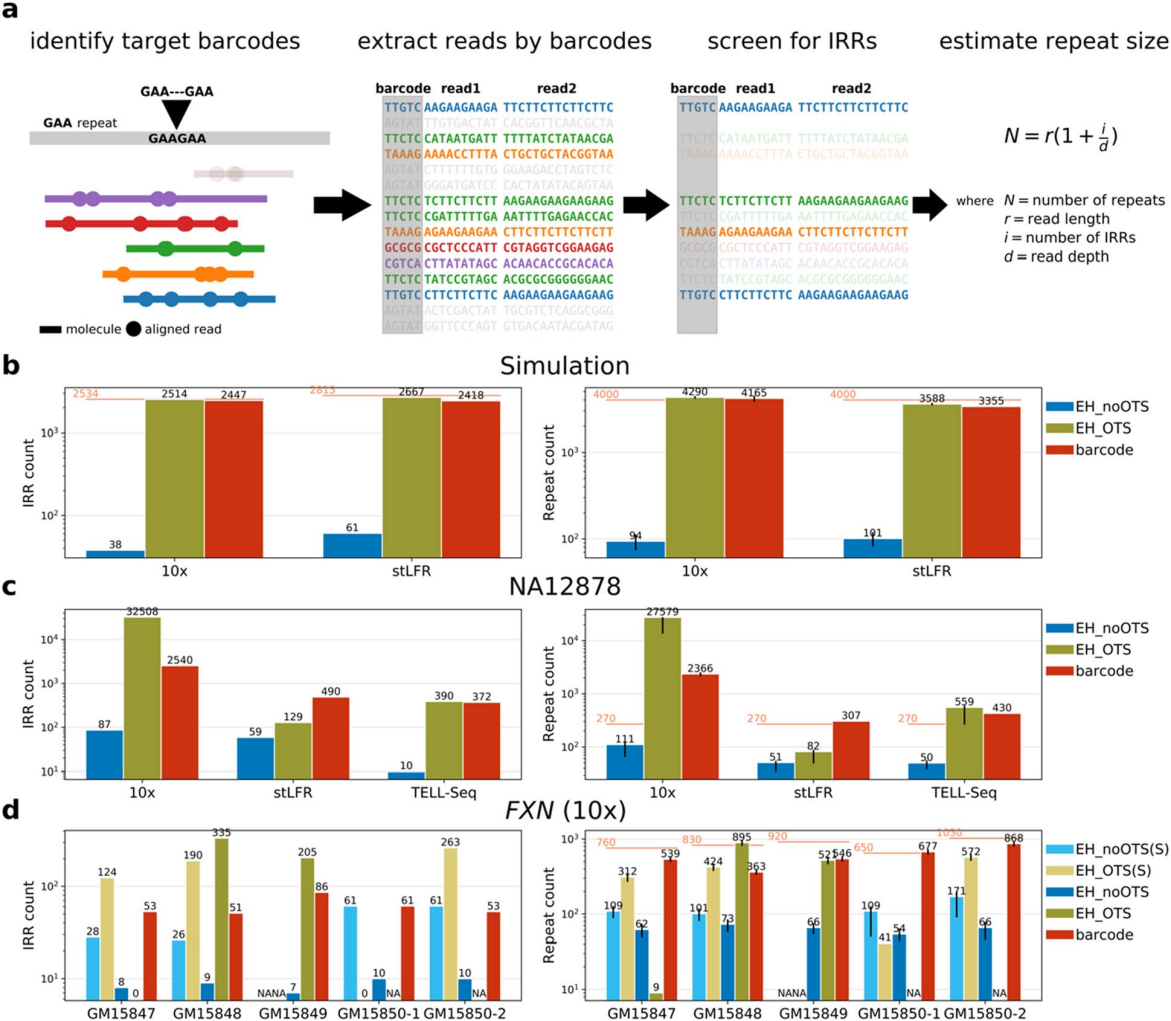
IRR extraction using barcodes in BLRS. (**a**) Steps in using barcodes for IRR extraction and STR size estimation. (**b–d**) IRR counts (left) and repeat count estimates (right) of the target loci within the three groups of datasets: (**b**) heterozygous ATTCT expansion in *ATXN10* 10 × and stLFR simulations; (**c**) homozygous larger-than-reference CCAT polymorphism in NA12878 from 10x, stLFR, and TELL-Seq BLRS platforms; and (**d**) *FXN* GAA expansions in 10 × data of four Coriell cell lines. The methods in comparison were EH without OTS (EH_noOTS, blue), EH with OTS (EH_OTS, olive), and barcode-based IRR extraction (barcode, red). For the *FXN* samples, EH results (with or without OTS) from standard Illumina data (EH_OTS(S), light blue and EH_noOTS(S), light olive) were also included. Only results of the expanded alleles in the samples were shown (therefore two separate tallies for the homozygous *FXN* GM15850 sample). “Expected” or “ground truth” IRR counts (for the simulations) and repeat counts were plotted as orange horizontal bars together with the exact numbers. Exact IRR or repeat counts were shown on top of each bar. Confidence intervals of the estimates reported by EH were shown as error bars. For the custom barcode-based method, the error bars reported for 10 × data corresponded to the range of estimates calculated independently using each of the two read lengths (see the Methods section). “NA” indicates that results were unavailable for certain samples because of a segmentation fault in EH runs.

To ascertain whether EH’s performance improves with barcode-retrieved IRR counts, we first identified barcodes from all linked reads that map to the target region and extracted all reads carrying the identified barcodes from the FASTQ files. We then screened these reads to detect IRRs composed of the target motif, and supplied the IRR counts, read length, and sequencing depth to the formula EH uses to impute STR sizes^14^ (see the Methods section; Fig. 1a).

By matching the identities of IRRs collected using barcodes against the “ground truth” determined from the simulated *ATXN10* data, we observed both high sensitivity (97% for 10x; 86% for stLFR) and specificity (100% for both 10 × and stLFR) in IRR extraction. All identified IRRs also originated from the haplotype that was simulated to contain the *ATXN10* RE. The EH repeat length estimates obtained from applying these barcode-derived IRR counts were close to the ground truth (96% for 10x; 84% for stLFR). EH analysis with OTS and barcode-retrieved IRRs yielded higher IRR counts (Fig. 1b; left panel) and much better repeat length estimates (Fig. 1b; right panel) compared to EH analysis without OTS.

Next, we genotyped a 1.1 kb CCAT STR (chr20:38194564-38194636, GRCh38) in the NA12878 BLRS data (Fig. 1c). This 76 base pair (bp) long locus in the reference genome was selected based on our analysis of the Nanopore long-read data of the same sample with Straglr^16^ and was independently confirmed to have a biallelic expansion (1050 and 1106 bp) from a high-quality haplotype-resolved assembly^17^. In both stLFR and TELL-Seq barcoded datasets, more IRRs were retrieved in comparison to EH analysis without OTS (Fig. 1c; left panel), which led to better size estimates (Fig. 1c; right panel), while results from EH analysis with OTS were either comparable to or poorer than those of the barcode-based analysis. Unexpectedly, we obtained abnormally high IRR counts and repeat sizes for NA12878 10 × data. Given that the sequencing depth of the 10 × data is similar to that of both stLFR and Tell-Seq, we could not determine the reason for this anomalous result, which was also reported by EH with OTS.

The 10 × *FXN* dataset had three heterozygous samples (GM15847-9) and one homozygous sample (GM15850). Using phasing results from the 10 × Long Ranger pipeline, we extracted IRRs (Fig. 1d; left panel) and calculated repeat sizes separately for each haplotype (Fig. 1d; right panel). As anticipated, all detected IRR-associated barcodes were assigned to the expanded haplotype in heterozygous and homozygous samples. The resulting IRR counts, however, led to slight under-sizing for all but one *FXN* allele in the homozygous sample. We also included the EH results of standard PCR-free Illumina data of the same Coriell cell lines (except GM15849) for comparison. Overall, barcode-based genotyping in BLRS data appeared to generate closer-to-expected repeat lengths for the *FXN* samples. We could not extract any IRRs from the 10 × *FMR1* samples (data not shown). This may be caused by under-representation of extremely GC-rich fragments as a result of PCR amplification in library construction for all the tested BLRS platforms.

**Figure 2:**
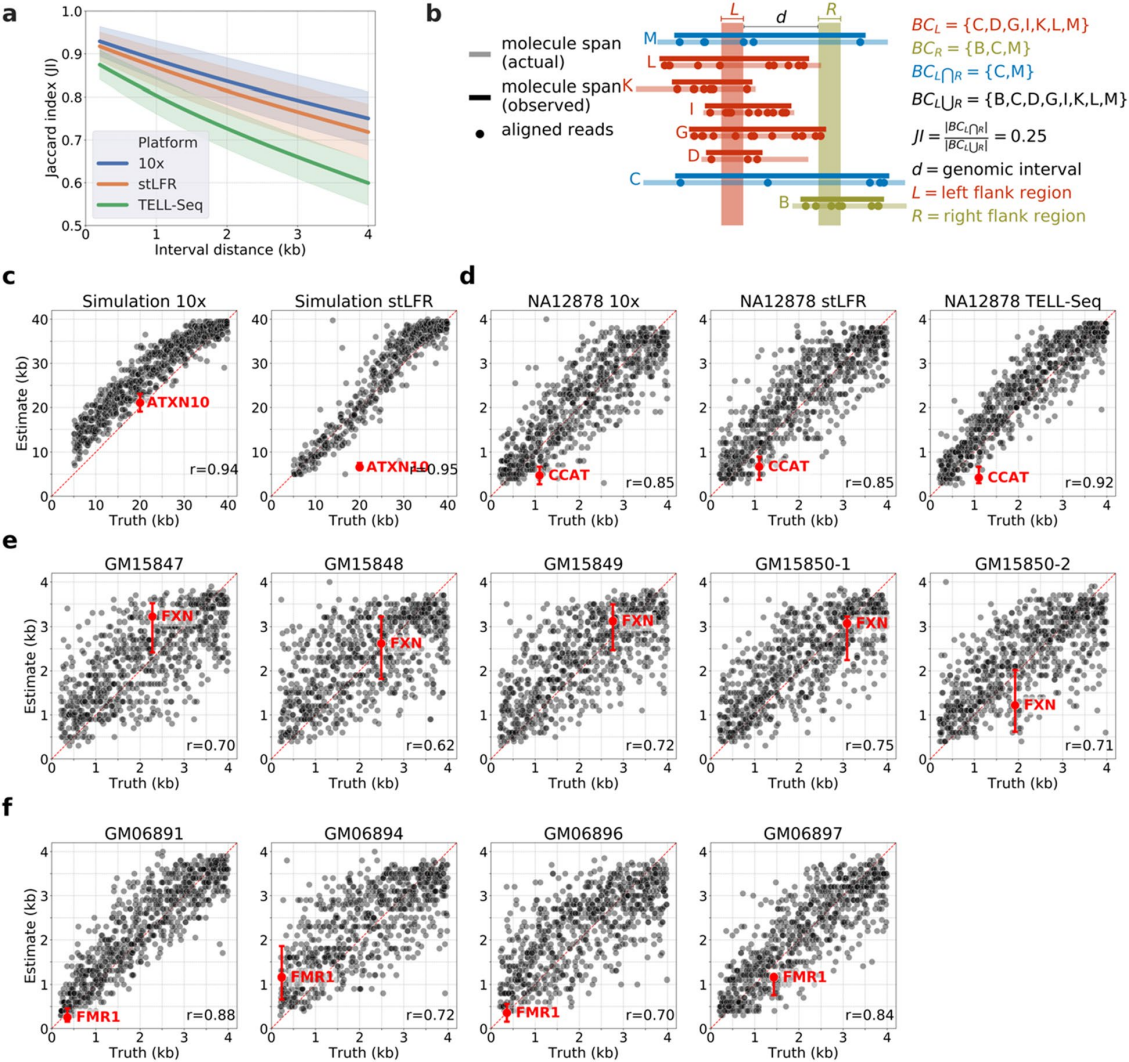
Size estimation of genomic intervals and STR loci using Jaccard index of barcode sharing in BLRS. (**a**) Inverse relationship between Jaccard index and genomic interval size observed in NA12878 of each of the three BLRS platforms. The colored bands correspond to the 95% confidence intervals for each platform. (**b**) Schematic of a hypothetical example illustrating the concept and terminology in computing the Jaccard index of barcode sharing for a given genomic interval. (**c**–**e**) Scatter plots of estimates (*y*-axis) vs. truths (*x*-axis) for ~ 700 arbitrary genomic intervals (black) and the target STR (red) in the simulation (**c**), NA12878 (**d**), *FXN* (**e**), and *FMR1* (**f**) datasets. Only estimates of the expanded allele at the target loci were shown. Confidence intervals of the estimates of the target loci were shown as red error bars. Dotted red diagonal lines were added to help visualize the amount of deviation of the estimates from the true values. “Truths” (*x*-axis) for the ~ 700 genomic intervals in all plots were calculated based on hg38 genomic coordinates. “Truth” for the target locus is the size of the *ATXN10* repeat we replaced the reference with in the modified genome to generate the simulated datasets (**c**); size of the CCAT allele we determined from the NA12878 assembly (**d**); sizes of the *FXN* (**e**) and *FMR1* (**f**) repeats in the Coriell samples according to on-line information of the respective cell lines (Table S1).

We also developed an alternative approach to genotyping STRs in BLRS data using a generic distance estimation method. Observing an inverse relationship between the Jaccard index (JI) of barcode-sharing and genomic distance in the NA12878 BLRS data (Fig. 2a), we generated size estimates of target loci by first creating a database storing counts of flanking barcodes and the resulting JI of varying genomic distances at a large number of random locations across the reference genome (Fig. 2b). We then performed the same computations for the target locus and searched among the interrogated genomic intervals for the closest profile in terms of flanking barcode counts and JI. The sizes of such closest-matched intervals formed the basis of our size estimate (see the Methods section).

We applied this methodology to all the datasets we used for IRR analysis (Fig. 2c–f). For each sample, we produced size estimates of the target STR and around 700 other randomly-chosen genomic loci to gain an overall performance benchmark of the method. For samples that are heterozygous for the target expansion (or homozygous but with different sizes) with phasing data available, the initial genome-wide profiling and target size estimates were performed on the two haplotypes separately. We observed a high overall correlation between estimates and “ground truth” from the random intervals with a Pearson correlation coefficient between 0.85 and 0.95 (Fig. 2c and d). The correlations weakened, however, when only barcodes belonging to a single haplotype were used (Fig. 2e and f). This drop in performance is anticipated as the coverage substantially decreased as phasing could not be achieved on all alignments. Nevertheless, comparing the result of the *ATXN10* allele in the 10 × simulation, for which a haplotype-specific estimate was attained, against that of the stLFR simulation, where phasing data were unavailable, it is clear that haplotype information is critical for accurate size estimation (Fig. 2a).

Size estimates were performed separately for individual haplotypes for the *FXN* 10 × samples, and the results were within reasonable ranges of “actual” sizes that were deduced from orthogonal analysis. The 1.1 kb “expansion” allele in NA12878 was under-sized, in contrast to the fairly good size estimate of the 20 kb *ATXN10* allele (Fig. 2d). We concluded that this methodology performs better for large genomic intervals (or STR expansions) over 1 kb. This is expected as the algorithm is premised on the stochastic bridging of genomic intervals by large DNA molecules, and the variability of such bridging within small intervals is limited.

We did not expect the JI method to yield accurate size estimates for the premutation (< 200 repeats) *FMR1* samples (GM06891, GM06894, and GM06896), as their allele sizes were smaller than its detection limit (Fig. 2f). Indeed, we observed a false-positive estimate (> 1 kb) for GM06894. Although the estimates for GM06891 and GM06896 happened to be close to the real allele sizes, over-estimates in the range of 100–500 bp were frequently observed for non-expanded reference-sized (< 50 bp) STRs (data not shown). Nevertheless, the full-mutation estimate in GM06897 (1.16 kb) was within 20% of the expected size (1.43 kb).

To compare the performance of the JI method against the IRR method, we additionally simulated 10 expansions ranging from 500 to 5000 bp in 8 known STR expansion disease genes and identified 13 loci from NA12878 that displayed expansions 1–3 kb in size over the reference genome (Table S2a and S2b). Out of the 8 genes in the 10 × and stLFR simulations, 7 were genotyped within 20% of the copy numbers simlulated using the IRR method in either simulation and 4 using the JI method. Interestingly both expansion alleles of the two homozygous cases, *GLS* and *RFC1*, matched the ground truths using either the IRR or the JI method in the 10 × simulation for which haplotype phasing was available and utilized (Fig.  S1). With the same criteria but using the assembly as the ground truth, 7 of the 13 NA12878 alleles were matched in either the stLFR and TELL-Seq data using the IRR method (10 × data excluded from calculation because of exorbitant IRR tallies as discussed above), compared to all 13 using the JI method (Fig. S2). We speculated that the JI method outperformed the IRR method on these loci because it does not require interrogation of read sequences which, in repeat loci of low purity, could lead to missed assignment of IRR to their intended targets. We envisage that both barcode-based methods can be deployed when there is no support evidence of either repeat-spanning reads or anchored IRR pairs from EH analysis, suggesting potentially an expansion event exists with a length well above the single short-read sequencing read or fragment length. The non-sequence-based JI method is a particularly useful complement to the IRR method when the target locus is located in low-coverage regions or in cases when IRR recruitment for the target locus fails, possibly because the expansion is composed of repeat interruptions or littered with artifacts resulting from amplification processes during the BLRS library preparation and sequencing steps.

Neither methodology introduced here (IRR or JI) is computationally expensive (Table S3). The most time-consuming barcode-based read extraction step in the IRR method and the genome-wide profile collection step in the JI method need to be performed only once for genotyping multiple target loci.

## Conclusions

We demonstrated that the long-range sequence information provided by BLRS barcodes can be leveraged to improve IRR detection and STR genotyping in conjunction with existing short-read genotypers such as EH. However, the dependence of current BLRS technologies on amplification steps in library preparation leads to severely diminished coverage for extremely GC-rich regions, thus making genotyping STR expansions of loci such as *FMR1* and *FMR2* very challenging or impossible. For these cases, we proposed and demonstrated the utility of an orthogonal approach bypassing sequence interrogation using barcode information to generate approximate size estimates of kb-scale expansions. Moreover, we demonstrated the value of haplotype phasing information of BLRS in segregating alleles in loci with biallelic REs. We expect that continued improvements in phasing algorithms would lead to more accurate genotype calls. Overall, our analysis showed that BLRS, despite some limitations discussed above, may have potential utility in STR genotyping and RE analysis.

## Methods

### Extracting IRR reads using barcodes

Alignments within 250 kb on either side of each target locus were examined to identify barcodes of DNA molecules that potentially carry the entire expanded repeat. Screening was performed to retain alignments that met the following conditions: (a) the alignment length is equal to the sequence length, and (b) properly paired as defined by SAMtools^18^. The start and end genomic coordinates of all retained alignments were stored for the associated barcodes. If phasing was performed and reported in the alignment records (e.g., by the 10 × Long Ranger pipeline), the haplotype assignment of each read was also transferred to the stored barcodes. The start and end of the sorted alignment coordinates of each barcode were then assigned as the spanning boundaries of the underlying DNA molecules. Only barcodes of molecules at least 1 kb long and within 10 kb of either boundary of the target locus were kept. Input reads in FASTQ format for the alignments were screened and all paired sequences containing the barcodes identified as described above were extracted for further screening for potential IRRs of the target locus. All the extracted read sequences were analysed with Tandem Repeat Finder^19^ (TRF) for STR detection. Reads with both mates composed entirely of the concatenation of the target repeat motif (with one in reverse-complement) were considered IRR pairs. In cases where only one of the paired reads was deemed entirely IRR, the other non-IRR mates were checked to see if the non-repeat portions could be mapped unambiguously to the up- or downstream region (500 bp) of the target locus using BLASTN^20^. The successful pairs were considered IRR anchors. Tallies of IRR (i.e., 2 × (IRR pairs + IRR anchors)) were segregated by haplotype and used for calculation of haplotype-specific repeat size estimates if haplotype assignments to barcodes were available.

### Calculation of repeat size using IRR counts

The following formula, interpreted from the original publication of EH^10,14^, was used for estimating repeat size:

$$ N = r\left( {{1 } + i/d} \right) $$

where *N* = estimated number of repeats, *r* = read length, *i* = number of IRRs, and *d* = read depth.

*r* was set to be 128 or 151 for 10 × reads since the first read consisted of the 16 bp barcode and 7 bp adaptor sequence. Sizes calculated from each *r* individually formed the lower and upper boundaries of the estimate. stLFR reads are 100 bp long. Different lengths were observed for individual TELL-Seq reads in both the FASTQ and BAM files, possibly due to various amounts of quality trimming. We used the maximum length 146 bp as *r* in the above formula for TELL-Seq samples. *i* is taken as the sum of double the number of IRR pairs and the number of IRR anchors. *d* is calculated as the mean of the median read depths of a 1 kb region 500 bp upstream and downstream of the target region. The purpose of adding margins was to avoid potential coverage abnormalities caused by mapping difficulties in the immediate vicinities of the STR regions.

### Determination of Jaccard index of barcode sharing

At a given genomic interval (*d*), we defined the left (*L*) and right (*R*) flanking regions as the 1 kb regions immediately upstream and downstream of the interval, respectively (Fig. 2b). Within 5 kb upstream and downstream of the flanking regions, we collected barcodes from all alignments and stored the start and end alignment coordinates sorted by barcode. Barcode identities were either available from the “BX” tags in the Long Ranger processed BAMs of 10 × and TELL-Seq samples or extracted from read names (portion after “#”) for stLFR samples. The span for each barcode was established as the first and last coordinates sorted numerically. Barcodes encompassing the left (*BC*_*L*_) or right (*BC*_*R*_) flanking regions were identified by overlapping each barcode span with the boundaries of each region (Fig. 2b). Jaccard index (*JI*) is calculated as the ratio of the number of shared (|*BC*_*L∩R*_|) to the total number of barcodes (|*BC*_*L*∪*R*_|) spanning either flanking region (Fig. 2b).

### Size estimate of genomic intervals using Jaccard index

Size estimate of a test genomic interval is derived from comparing its (*BC*_*L*_, *BC*_*R*_, *JI*) against the same tuples calculated from a large number of random, pre-determined intervals (database). The Euclidean distance between the test tuple and each tuple in the database was computed and the sizes of the topmost similar (*E*) intervals with Euclidean distances less than a preset value, *F*, were kept (progressive trials of small-to-large values of *F* may need to be performed to identify *E* similar intervals from the database). The median and the inter-quartile range of the sizes of the retained intervals were used as the estimate and range of the prediction. To generate the databases, we randomly picked 50,000 positions in the reference autosomes excluding sequence gaps and regions that may pose challenges to alignment, such as segmental duplications and peri-centromeric regions. For the NA12878 and *FXN* samples, we interrogated intervals from 200 to 4000 bp at a step-size of 100 bp at each position. For the simulation samples, because the expansion allele is larger, interval sizes from 5 to 40 kb at a step-size of 500 bp were used instead. *E* was set to be 200; *F* was set as 4 for NA12878 and *FXN* samples but increased to 200 for the large allele in the simulation samples as the differences between the tuples were much increased.

## Supplementary Information

Supplementary Information.

## Data Availability

10 × Genomics and MGI stLFR NA12878 sequences and alignments were downloaded from ftp://ftp-trace.ncbi.nlm.nih.gov/ReferenceSamples/giab/data/NA12878/10Xgenomics_ChromiumGenome_LongRanger2.0_06202016 and ftp://ftp-trace.ncbi.nlm.nih.gov/ReferenceSamples/giab/data/NA12878/stLFR/ respectively. Universal Sequencing Technology TELL-seq NA12878 sequences were downloaded from the Sequence Read Archive under the accession SRX7264480. Software developed for this manuscript is available from https://github.com/bcgsc/link_str^21^ under the license GNU General Public License version 3 (GPLv3) and archived under 10.5281/zenodo.5428975^22^.
