# Supplementary material for: Linked-read sequencing for detecting short tandem repeat expansions
Source: Sci Rep. 2022 Jun 7;12:9352. doi: 10.1038/s41598-022-13024-4 (PMC9174224; doi:10.1038/s41598-022-13024-4)
Supplement: Supplementary file 1 — Supplementary Information. [file 41598_2022_13024_MOESM1_ESM.pdf]

Supplementary Information: Linked-read sequencing for detecting short tandem repeat expansions

Readman Chiu[1*], Indhu-Shree Rajan-Babu[2,3*], Inanc Birol[1,2], Jan M. Friedman[2,4]

[1]Canada's Michael Smith Genome Sciences Centre, BC Cancer, Vancouver, BC V5Z 4S6, Canada

[2]Department of Medical Genetics, University of British Columbia, Vancouver, BC V5Z 4H4, Canada

[3]Department of Medical and Molecular Genetics, King's College London, Strand, London WC2R 2LS, UK

[4]BC Children's Hospital Research Institute, Vancouver, BC V5Z 4H4, Canada

*contributed equally

Corresponding author

Inanc Birol: ibirol@bcgsc.ca

10x sequencing of Coriell samples

Four and six reference lymphoblastoid cell lines with GAA- and CGG-repeat expansions in the *frataxin* (*FXN*) and *fragile X mental retardation 1* (*FMR1*) genes, respectively, were obtained from the Coriell Cell Repository (Camden, New Jersey, United States; https://www.coriell.org/). The *FXN* and *FMR1* reference samples, their genotypes (normal/NL, premutation/PM, and full-

mutation/FM), and repeat lengths reported by Coriell are shown in Supplemental Table 1. High molecular weight genomic DNA derived from the cell lines was used for library preparation with the Chromium Genome Reagent Kit (v2 Chemistry). Paired-end whole-genome sequence (WGS) reads of 150 base pairs were generated on an Illumina HiSeq X instrument and processed using the Long Ranger™ pipeline (v2.2.2) from 10x Genomics. The generated reads were mapped to the hg19 human reference genome build.

Table S1. *FMR1* and *FXN* genotypes and allelic repeats lengths of Coriell samples used for 10x linked-read whole-genome sequencing. The two normal samples, GM06889 and GM06895, were not analyzed using the barcode-based methods presented in the manuscript. On-line information to the cell lines is accessible through the hyperlinks under "Coriell ID".

Coriell ID	Actual genotype	Gene	Repeat motif	Repeat size (copy number)
GM15847	NL/FM	*FXN*	GAA	NL/760
GM15848	NL/FM	*FXN*	GAA	NL/830
GM15849	NL/FM	*FXN*	GAA	NL/920
GM15850	FM/FM	*FXN*	GAA	650/1030
GM06889	NL/NL	*FMR1*	CGG	23/30
GM06891	PM	*FMR1*	CGG	118
GM06894	NL/PM	*FMR1*	CGG	30/78
GM06895	NL	*FMR1*	CGG	23
GM06896	NL/PM	*FMR1*	CGG	23/95-120-140
GM06897	FM	*FMR1*	CGG	477

Table S2a. Information of modified loci in 10x and stLFR simulations used for method comparison.

Gene	Chromosome	Motif	Size (bp)	Copy number	Zygosity	Sex
GIPC1	19	CCG	500	167	het	male
ATXN8OS	13	CTG	750	250	het	male
FMR2	X	CCG	1000	334	hemi	male
C9orf72	9	GGCCCC	1500	250	het	male
SAMD12	8	TGAAA	2000	400	het	male
GLS	2	GCA	2500	834	homo	male
GLS	2	GCA	3000	1000	homo	male
RFC1	4	AAGGG	3500	700	homo	male
RFC1	4	AAGGG	4500	900	homo	male
NOP56	20	GGCCTG	5000	834	het	male

Table S2b. Information of expanded (relative to reference genome) loci in NA12878 assembly used for method comparison.

Locus	Motif	Reference Size (bp)	Assembly sizes (allele1/allele2 bp)
chr11:133354203-133354353	TGG	151	1715/2790
chr11:92705289-92706006	GTG	718	2437/2411
chr13:112972218-112972863	TGGA	646	1612/1612
chr13:21725858-21726151	CACCAC	294	2990/3069
chr18:22231934-22232859	TCCA	926	2207/2331
chr18:77252855-77253057	CAC	203	1496/506
chr4:5706294-5706480	ATAG	187	2456/3136
chr4:7724106-7725052	GTG	947	1269/1627
chr4:8294583-8295012	TCCA	430	1640/1640
chr6:3221957-3222326	CAC	370	1631/1600
chr6:834343-834462	TCCT	120	1843/1876
chr7:141336723-141337391	CCA	669	1962/1515
chr7:98810989-98811637	GTG	649	3389/3385

het: heterozygous; homo: homozygous; hemi: hemizygous

Table S3. Computing benchmarks of both IRR and JI methods. Computing was performed on an Intel Xeon Gold 6254 3.10 GHz 144-core machine running Centos 7.6.

Method	Step	Time	Memory (Peak)
IRR	1. Extract reads based on barcodes mapped to target region (extract_irr.py)	36m:04s	550.79 MB
	2. Identify IRR reads from extracted read set (id_irr.py)	4m:53s	1.73 GB
	3. Determine repeat size based on IRR counts, read length, and coverage (get_irr_size.py)	14.35s	82.71 MB
	Benchmarking based on genotyping 13 loci on NA12878 stLRF data (3.34B reads)		
JI	1. Generate database of JI profiles for varying genomic intervals at random locations (model_span.py)	14m:11s	32.25 GB
	2. Match JI profile of target region against database to estimate target's genomic distance (estimate_span.py)	18.645s	964.44 MB
	Benchmarking based on collecting JI profiles from 50,000 locations at distances from 200 to 10,000 at a step-size of 100 bp (step 1) and generating estimates for 13 loci (step 2) on NA12878 TELL-Seq data (1.11B reads).<br>Step 1 was run with multi-processing using 42 parallel processes.		

Fig. S1. A comparsion of genotyping performance on simulated STR expansions in BLRS data between ExpansionHunter (EH), barcode-based in-repeat reads (IRR), and Jaccard index (JI) methods. Black vertical bars indicate repeat counts of loci in reference genome. Orange vertical bars indicate repeat counts of simulated alleles. EH_noOTS: EH analysis without off-target sites (OTS) and EH_OTS: EH analysis with OTS.

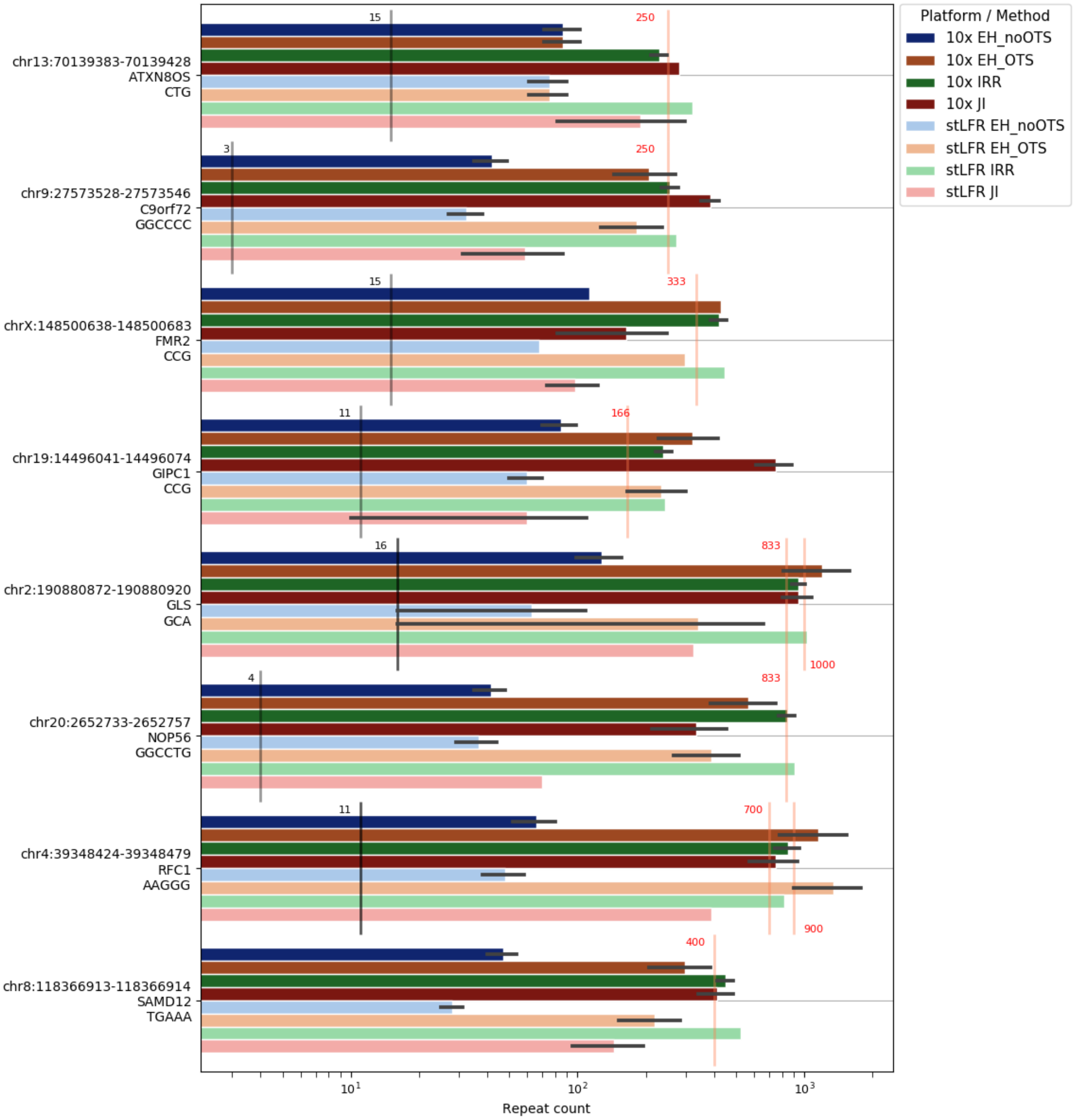

Fig. S2. A comparsion of genotyping performance on STR expansions, relative to the human genome and verified by *de novo* assembly, in NA12878 BLRS data between ExpansionHunter (EH), barcode-based in-repeat reads (IRR), and Jaccard index (JI) methods. Black vertical bars indicate repeat counts of loci in reference genome. Orange vertical bars indicate repeat counts of alleles determined from sequence assembly. “NA” indicates results not available because of failed software run or software’s failure to genotype specific locus. EH_noOTS: EH analysis without off-target sites (OTS).

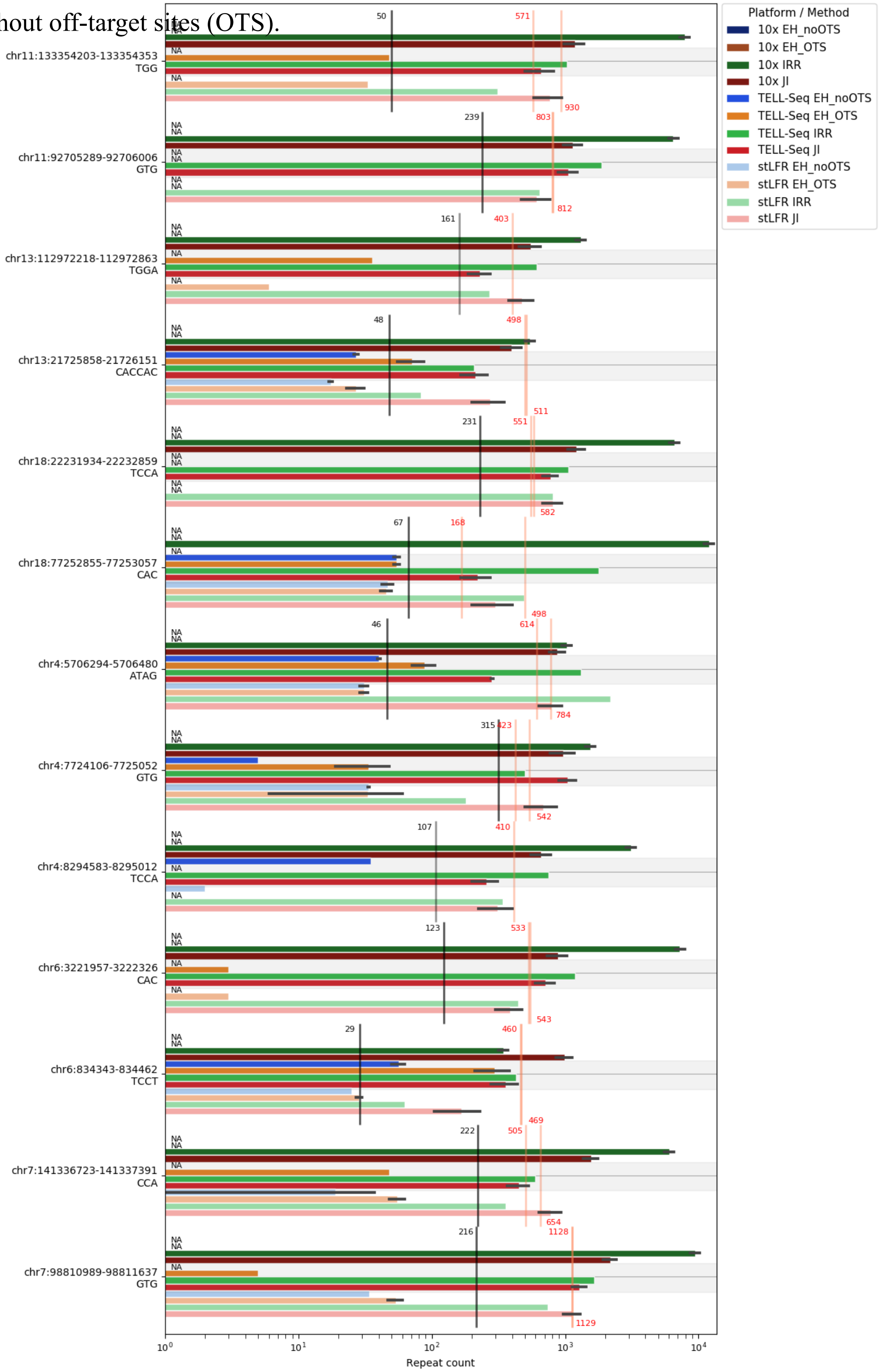

10x and stLFR simulation

10x and stLFR WGS with repeat expansions were simulated using LRSim[1] (v1.0) and stLFR read simulator[2], respectively. The *ATXN10, ATXN8OS, GIPC1, GLS, NOP56, RFC1, C9ORF72, SAMD12,* and *FMR2* repeat loci in the hg38 human reference genome were modified to contain expanded stretches of repeats (shown in Table S2a) using reform[3]. The modified and unmodified reference FASTA files were processed using LRSim's simulateLinkedReads.pl script in -r (reference) mode:

```
perl simulateLinkedReads.pl -r $modFASTA -p $output_prefix -7 0 -0 0 -z 16

perl simulateLinkedReads.pl -r $unmodFASTA -p $output_prefix -7 0 -0 0 -z 16
```

"clean" FASTA files from the modified and unmodified reference FASTA were used as inputs with the simulateLinkedReads.pl and stLFRSim to generate 10x and stLFR reads as follows:

```
perl simulateLinkedReads.pl -g hg38.hap.0.clean.fasta,hg38.hap.1.clean.fasta -p $output_prefix
-7 0 -0 0 -z 16 -x 350 -i 500 -s 50 -o

stLFRSim  --ref hg38.hap.1.clean.fasta
          --o_prefix $output_prefix
          --lr_length_distribution lr_length_dis.txt
          --pe_num_distribution pe_num_dis.txt
          --if_lenth_distribution pe_length_dis.txt
          --readpair_num 225000000

stLFRSim  --ref hg38.hap.0.clean.fasta
          --o_prefix $output_prefix
          --lr_length_distribution lr_length_dis.txt
          --pe_num_distribution pe_num_dis.txt
          --if_lenth_distribution pe_length_dis.txt
          --readpair_num 225000000
```

The generated paired-end 150 base pair 10x reads from the modified and unmodified sequences were concatenated and processed using Long Ranger:

```
longranger wgs  --id=sim${i}
                --fastqs=$fq
                --reference=$PATH/reference_genomes/H_sapiens/10X-Genomics/refdata-
GRCh38-2.1.0
                --localcores=30
                --localmem=120
                --vcmode=freebayes
                --sex=female
```

The generated paired-end 100 base pair stLFR reads from the modified and unmodified sequences were concatenated and mapped to the hg38 reference genome using BWA-MEM[4], duplicates in the BAM file marked with Picard[5], and indexed using SAMtools[6].

EH analysis with OTS

The EH variant catalog file used for *ATXN10* analysis (hg38) is:


```
{
    "OffTargetRegions": [
        "chrX:9791969-9792159"
    ],
    "RepeatId": "ATXN10-withOTS",
    "RepeatUnit": "ATTCT",
    "TargetRegion": "chr22:45795355-45795424"
}
```


The EH variant catalog file used for *FXN* analysis (hg19) is:


```
{
    "OffTargetRegions": [
        "chr1:102123410-102123560",
        "chr13:102813926-102814076",
        "chr2:221410907-221411057",
        "chr5:126583029-126583179",
        "chrX:51364391-51364541"
    ],
    "RepeatId": "FXN-withOTS",
    "RepeatUnit": "GAA",
    "TargetRegion": "chr9:71652203-71652220"
}
```


The EH variant catalog file used for *FMR2* analysis (hg38) is:


```
{
    "OffTargetRegions": [
        "chr17:32142451-32142503",
        "chr19:10871563-10871651",
        "chr2:86914348-86914529",
        "chr7:100694312-100694385",
        "chrX:19990922-19990974",
        "chrX:67546512-67546566",
        "chrX:150983398-150983456"
    ],
    "RepeatId": "FMR2",
    "RepeatUnit": "CCG",
    "TargetRegion": "chrX:148500638-148500683"
}
```


The EH variant catalog file used for *GIPC1* analysis (hg38) is:


```
{
```

“OffTargetRegions”: [
“chr17:32142451-32142503”,
“chr19:10871563-10871651”,
“chr2:86914348-86914529”,
“chr7:100694312-100694385”,
“chrX:19990922-19990974”,
“chrX:67546512-67546566”,
“chrX:150983398-150983456”
],
“RepeatId”: “GIPC1”,
“RepeatUnit”: “CCG”,
“TargetRegion”: “chr19:14496041-14496074”
}

The EH variant catalog file used for *GLS* analysis (hg38) is:

{
"OffTargetRegions": [
"chr16:73546662-73546734",
"chr17:10995712-10995773",
"chr17:51831668-51831732",
"chr18:55586153-55586230",
"chr19:45770206-45770266",
"chr3:149766818-149766881",
"chr7:7673311-7673371",
"chrX:67545316-67545385"
],
"RepeatId": "GLS",
"RepeatUnit": "GCA",
"TargetRegion": "chr2:190880872-190880920"
}

The EH variant catalog file used for *NOP56* analysis (hg38) is:

{
"OffTargetRegions": [
"chr5:146459014-146459174"
],
"RepeatId": "NOP56",
"RepeatUnit": "GGCCTG",
"TargetRegion": "chr20:2652733-2652757"
}

The EH variant catalog file used for *RFC1* analysis (hg38) is:

{
"OffTargetRegions": [

"chr10:25874364-25874549",
"chr10:100315011-100315116",
"chr11:63257658-63257763",
"chr21:39583818-39584123",
"chr5:79180390-79180505",
"chr5:79180514-79180629",
"chrX:48280812-48280912"
],
"RepeatId": "RFC1",
"RepeatUnit": "AAGGG",
"TargetRegion": "chr4:39348424-39348479"
}

The EH variant catalog file used for *SAMD12* analysis (hg38) is:

{
"OffTargetRegions": [
"chr21:34718631-34718684",
"chr3:151368620-151368760",
"chr4:114319040-114319089",
"chr4:122718712-122718762",
"chr6:157534533-157534600",
"chr8:139313899-139313962",
"chr9:78008566-78008623"
],
"RepeatId": "SAMD12",
"RepeatUnit": "TGAAA",
"TargetRegion": "chr8:118366913-118366914"
}

The EH variant catalog file used for *C9ORF72* analysis (hg38) is:

{
"OffTargetRegions": [
"chrX:154390486-154390564"
],
"RepeatId": "C9ORF72",
"RepeatUnit": "GGCCCC",
"TargetRegion": "chr9:27573528-27573546"
}

Generation of OTS: To identify the off-target sites, we simulated 100 or 150 bp reads entirely composed of the “motif of interest” and mapped these reads to the reference genome (hg19 or

hg38) using bwa mem. The mapping coordinates of the reads were extracted from the sorted BAM files using bedtools and were provided as off-target sites to EH.

Illumina Coriell genomes

The Illumina Coriell genomes[7] analysed in this study can be accessed in the European Genome-phenome Archive repository: https://www.ebi.ac.uk/ega/datasets/EGAD00001003562.

References

1. Luo, R., Sedlazeck, F. J., Darby, C. A., Kelly, S. M. & Schatz, M. C. LRSim: A Linked-Reads Simulator Generating Insights for Better Genome Partitioning. *Comput. Struct. Biotechnol. J.* **15**, 478–484 (2017).
2. GitHub - BGI-Qingdao/stLFR_reads_sim: [Develeping] Reads Simulator of stLFR. *GitHub* https://github.com/BGI-Qingdao/stLFR_reads_sim.
3. reform: Modify Reference Sequence and Annotation Files Quickly and Easily – Genomics Core at NYU CGSB. https://gencore.bio.nyu.edu/reform/.
4. Li, H. Aligning sequence reads, clone sequences and assembly contigs with BWA-MEM. (2013).
5. Picard Tools - By Broad Institute. http://broadinstitute.github.io/picard/.
6. Li, H. *et al.* The Sequence Alignment/Map format and SAMtools. *Bioinformatics* **25**, 2078–9 (2009).
7. Dolzhenko, E. *et al.* Detection of long repeat expansions from PCR-free whole-genome sequence data. *Genome Res* **27**, 1895–1903 (2017).
